# A phase I study on combined therapy with proton-beam radiotherapy and *in situ* tumor vaccination for locally advanced recurrent hepatocellular carcinoma

**DOI:** 10.1186/1748-717X-8-239

**Published:** 2013-10-16

**Authors:** Masato Abei, Toshiyuki Okumura, Kuniaki Fukuda, Takayuki Hashimoto, Masahiro Araki, Kazunori Ishige, Ichinosuke Hyodo, Ayae Kanemoto, Haruko Numajiri, Masashi Mizumoto, Takeji Sakae, Hideyuki Sakurai, Junko Zenkoh, Gerelchuluun Ariungerel, Yu Sogo, Atsuo Ito, Tadao Ohno, Koji Tsuboi

**Affiliations:** 1 Department of Gastroenterology, Faculty of Medicine, University of Tsukuba, 1-1-1 Tennohdai, Tsukuba, Ibaraki 305-8575, Japan; 2 Proton Medical Research Center, Faculty of Medicine, University of Tsukuba, 1-1-1 Tennohdai, Tsukuba, Ibaraki 305-8575, Japan; 3 Department of Gastroenterology, Ibaraki Prefectural Central Hospital, 6528 Koibuchi, Kasama, Ibaraki 309-1793, Japan; 4 Human Technology Research Institute, National Institute of Advanced Industrial Science and Technology (AIST), Central 6, 1-1-1 Higashi, Tsukuba, Ibaraki 305-8566, Japan; 5 Faculty of Science and Engineering, Waseda University, 3-4-1 Okubo, Shinjuku-ku, Tokyo, and Cell-Medicine, Inc, 2-1-6-C-B-1 Sengen, Tsukuba, Ibaraki 305-0047, Japan

**Keywords:** Proton-beam radiotherapy, Hepatocellular carcinoma, Immunotherapy, Immunoadjuvant

## Abstract

**Background:**

Proton-beam radiotherapy (PBT) has been shown to be effective to hepatocellular carcinoma (HCC) as a nonsurgical local treatment option. However, HCC still remains as one of the most difficult cancers to be cured because of frequent recurrences. Thus, methods to inhibit the recurrence need to be explored. To prevent the HCC recurrence, we here report on a prospective phase I study of *‘in situ’* tumor vaccination using CalTUMP, a newly developed immunoadjuvant consisting of BCG extract bound to hydroxyapatite and microparticulated tuberculin, following local PBT for HCC.

**Methods:**

Patients with locally advanced recurrent HCC, which had been heavily pretreated with various treatments, were enrolled. PBT was performed with the conventional method to the target HCC. Subsequently, CalTUMP was injected into the same irradiated-tumor three times at one-week intervals. Three dose-levels of CalTUMP (1/10, 1/3, and 1/1) were administered to 3 patients each. Vital signs, blood samples, ultrasound, and computed tomographic scans were monitored to evaluate the safety.

**Results:**

Three intratumoral injections of CalTUMP following PBT (median dose: 72.6 GyE) were accomplished in 9 patients. Transient low-grade fever and minor laboratory changes were observed in 7 patients after CalTUMP injections. No other treatment-related adverse events were observed. Median progression-free survival was 6.0 months (range: 2.1-14.2) and 4 patients were progression-free for more than 1 year.

**Conclusions:**

Intratumoral injection of CalTUMP following PBT was feasible and safe in patients with heavily pre-treated HCC. Further clinical studies to evaluate the efficacy of this *in situ* tumor vaccination are warranted.

## Background

Hepatocellular carcinoma (HCC) is the second leading cause of cancer-related deaths with approximately 750,000 new cases reported per year in the world [1,2]. The majority of HCC occurs in patients who developed liver cirrhosis secondary to chronic hepatitis B or C infections, alcohol abuse, or nonalcoholic steatohepatitis. In the past few decades, multimodal treatments of HCC, including surgical hepatectomy, radio-frequency ablation (RFA), transarterial chemoembolization (TACE), liver transplantation, and a molecular target-drug, sorafenib, etc. [3,4], have much progressed with consensus guidelines published by several organizations [5-7].

Despite these advances in the treatment, HCC still remains as one of the most difficult cancers to be cured, because multiple recurrences of the tumor are quite frequent [3-7]. Thus, methods that can effectively prevent the recurrence need to be explored rigorously. In addition, in Japan where approximately 75% of HCC are caused by hepatitis C virus infected during 1950′ to 80′, many HCC patients are now aged more than 75 years old and have limited treatment options or often cannot receive treatments recommended by the guidelines.

Radiotherapy (RT) had not been successful for HCC until late 1980′s, because only insufficient doses (<30 Gy) could be applied to the cirrhotic liver to avoid fetal radiation-induced liver disease (RILD). Proton beam, unlike conventional X-ray, forms a unique Bragg peak ionization that enables ‘tumor-targeted irradiation’ [8]. Based on this unique property, we have introduced proton beam therapy (PBT) for HCC since 1983, in collaboration with the High Energy Accelerator Research Organization at Tsukuba, and demonstrated the first evidence of curative yet safe radiotherapy for HCC [9]. Subsequently, we observed an excellent 5-year local tumor control rate of 87% and a 5-year overall survival of 24% in the first 168 HCC patients treated with PBT [10]. Patients with solitary HCC and Child-A liver function were associated with a good 5-year survival of 53.5% [10], which was comparable to the results of surgical resection [11]. We then opened an in-house PBT facility at Tsukuba University Hospital in 2001, and could demonstrate its excellent local tumor control rate (83%) and further improved 5-year survival rate (44.6%) in HCC patients [12]. Since PBT is safe and has limited effects on liver function [13], we especially recommend it to aged HCC patients [14] who prefer not to take the risk of surgery or to patients with limited treatment options due to various extrahepatic complications [15] or poor liver function [16]. We have also reported on its excellent efficacy for large HCC (> 5 cm) [17], HCC with portal vein tumor thrombosis (PVTT) [18], HCC adjacent to porta-hepatis [19] or alimentary tracts [20]. In addition, PBT can be further intensified by hypofractionation [21] and can be repeated safely [22]. Following these preceding clinical data of ours, a high efficacy of PBT for HCC has been confirmed by other newly developed PBT facilities [23-26]. The recent increase of PBT facilities worldwide may indicate that more HCC patients, especially elderly patients or those with complications, will be treated by PBT in the near future.

However, HCC patients who receive PBT, as well as those receiving other therapies, are not free from the high incidence of recurrences. In fact, the intra-hepatic extra-field recurrence following PBT is frequent: almost 50% at 1 year and 85% after 5 years [11,13]. In spite of additional localized therapies, most patients eventually suffer fatal hepatic failure due to repeated multiple recurrences. Therefore, prevention of intra-hepatic recurrence is clearly the most critical issue in improving the survival of patients with HCC.

One promising option in preventing recurrence is tumor immunotherapy. We have previously reported on a phase II randomized clinical trial in which we demonstrated that autologous formalin-fixed tumor vaccine (AFTV) made from resected tumor tissue significantly improved both overall and event-free survivals after surgery [27]. Although the results strongly suggest that AFTV is effective in preventing the recurrence of HCC, treatment availability is limited by the fact that a certain volume of autologous cancer tissue is required to produce the vaccine [28]. To overcome this limitation, we postulated that utilizing *in vivo* tumor tissue following local treatments, such as RFA or radiation, would enable us to induce a systemic immune response against the tumor.

Based on this idea, we report here the first clinical trial to test the efficacy of “*in situ* vaccination” approach using hydroxyapatite (HA; Ca_10_(PO_4_)_6_(OH)_2_) immune adjuvant injected into the tumor tissues, which were pretreated with the potent PBT. Our primary endpoints was to confirm the safety and the secondary endpoint was to evaluate the efficacy of PBT followed by direct intratumoral injection of HA adjuvant in patients with HCC. This novel approach combining confocal radiotherapy and systemic immunotherapy complements the drawbacks of each treatment, resulting in a more effective way to treat solid malignant neoplasms.

## Methods

### Study design

**Figure 1:**
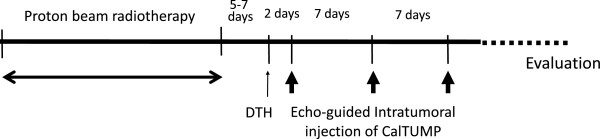
**Treatment schedule.** One treatment course comprises of proton beam radiotherapy (PBT) and 3 echo guided injections of CalTUMP every week. Delayed hypersensitivity test (DTH) using CalTUMP was performed 5–7 days after PBT. The first intratumoral injection of CalTUMP was performed 48 hours after DTH. The follow-up period was more than 1 year.

A prospective one-arm Phase I clinical trial was designed to evaluate the safety and efficacy of PBT followed by echo-guided direct intratumoral injection of a newly developed immunoadjuvant named “CalTUMP”, an HA adjuvant, in patients with HCC as illustrated in Figure 1. HA adjuvant dosage was increased incrementally as indicated in Table 1.

**Table 1:** Characteristics of patients enrolled in the study

**No**	**Age/gender**	**Child-Pugh (Score)**	**Size and number of tumors**		**KPS (%)**	**PBT dose/frac**	**Dose of Cal-TUMP**
			**Location (Size)**	**Number (LN)**			
1	73/M	A (5)	S4/5/8 (110 mm), S5/6 (8 mm), Lymph node (10 mm)	3 (1)	90	74 GyE/37f	1/10
2	55/M	A (6)	S3 (34 mm), S4 (58 mm, 8 mm), S8 (10 mm)	4 (0)	90	72 GyE/37f	1/10
3	52/M	A (5)	right lobe (110 mm), S3 (44 mm, 70 mm)	3 (0)	90	72.6 GyE/22f	1/10
4	63/M	A (5)	S1 (26 mm, 18 mm), portal region (19 mm)	3 (0)	90	72.6 GyE/22f	1/3
5	65/M	B (8)	S4 (87 mm), S5/7/8 (10 mm × 4)	5 (0)	90	52.8 GyE/16f	1/3
6	65/M	A (5)	S5 (54 mm), S1 (27 mm), S4 (8 mm) lateral segment of left lobe (12 mm), Lymph node (12 mm)	5 (1)	90	87.6 GyE/27f	1/3
7	59/M	A (6)	S5/8 (20 mm)	1 (0)	100	72.6 GyE/22f	1/1
8	72/M	A (5)	In the vicinity of IVC (46 mm)	1 (0)	90	72.6 GyE/22f	1/1
9	71/F	A (5)	S8 (14 mm, 14 mm)	2 (0)	100	72.6 GyE/22f	1/1

(LN: Lymph node, KPS: Karnofsky performance status, PBT: Proton beam radiotherapy, Dose.frac: Total dose/fraction number).

### Patient selection

Subjects were chosen from recurrent cases of locally advanced HCC who still retained their hepatic function. Eligibility criteria were as follows: 1) A pathological or radiological diagnosis of HCC. 2) Locally advanced HCC that recurred after standard therapy, with indications for additional PBT. 3) Quantifiable tumor size. 4) A life expectancy >6 months. 5) Adequate hepatic function to undergo PBT. 6) Adequate bone marrow function to undergo PBT. 7) Normal renal function (Serum creatinine < 1.5 mg/dL). 8) Karnofsky Performance Scale (KPS) 80% or higher. 9) Aged between 20 and 80. 10) Acknowledges the diagnosis and has given written informed consent. 11) Patients whose follow-up is possible at the University Hospital of Tsukuba or its affiliated hospitals.

The exclusion criteria were as follows: 1) History of malignancy other than HCC within the last 5 years. 2) A history of autoimmune disease. 3) Human immunodeficiency virus (HIV) infection. 4) Presence of complications including hematologic disorders or bleeding tendencies, which could disrupt protocol requirements. 5) Uncontrolled disease including serious infections, and cardiac or psychiatric disorders. 6) Presence of Grade 3 or higher bone marrow dysfunction as specified in the Common Terminology Criteria for Adverse Events (CTCAE v3.0) [29]. 7) Use of antineoplastics or corticosteroids within the last 4 weeks, systemic radiotherapy, or biological therapy. 8) Pregnant or intent to become pregnant. 9) Tuberculosis or skin reaction to CalTUMP exhibiting an induration >2 cm in the delayed hypersensitivity test. 10) Patients with conditions deemed unsuitable for inclusion in this study by the physicians in charge.

### Proton beam irradiation

PBT was conducted based on the computed tomography (CT) images taken at 5-mm intervals at the treatment site. A booster synchrotron generating 250 MeV proton beams at the Proton Medical Research Center (PMRC) was used. Dose distribution and settings for the collimator configuration, bolus, and range-shifter thickness were determined from the treatment plan. The relative biological effectiveness (RBE) of the PBT was assumed to be 1.1 [30]. Clinical target volume (CTV) was defined as the area encompassing the tumor on CT scans. Planned target area was defined as the CTV area plus a 10-mm margin. Two treatment protocols of 72.6 GyE in 22 fractions, and 74 GyE in 37 fractions were applied. The total dose and fractions for each patient are shown in Table 1.

### Immunoadjuvant CalTUMP

A calcium phosphate solution (RSM) supersaturated with respect to HA was prepared by mixing a 24.84 volume of Ringer’s solution (Fuso Pharmaceutical Industries, Ltd, Osaka, Japan) and a 3.72 volume of glucose-L-lactate-phosphate buffered saline (Ajinomoto Pharmaceuticals, Co. Ltd., Tokyo, Japan), with a 0.287 volume of 7% sodium bicarbonate (Otsuka Pharmaceutical Factory, Ltd., Naruto, Kochi, Japan). The RSM solution was further mixed with a 1/10th volume of ethanol and incubated at 50°C overnight. Resulting HA nanoparticles and their aggregated precipitates were collected via centrifugation at 1,200G for 15 min, and then washed with RSM solution. One ml of BCG extract was prepared by adding alcohol to 24 mg of freeze-dried BCG vaccine (Japan BCG Laboratory, Tokyo, Japan). The packed HA was resuspended in RSM solution and adjusted to a 3.4%(v/v), and a 10%(v/v) BCG extract was added. The mixture was incubated at 37°C for one day to coprecipitate BCG extract with HA on the HA nanoparticles. The resulting HA were collected, washed, and resuspended in RSM solution. Tuberculin microparticles (TUMP) were prepared according to a previously described method using 125 ng of PPD instead of 10^6^ U of IL-2 [28].

Dose of CalTUMP in this study was defined as follows; concentration of 1/1 corresponding to dose-3 was constructed with HA loaded with BCG extract at 3.2%(v/v, as the packed volume), TUMP at (as PPD) 125 mg/mL, and 0.01% human serum albumin in RSM solution. The final suspension contained 2.23 mg/ml of calcium ion as quantified by inductively coupled plasma-atomic emission spectroscopy. CalTUMP was diluted with 0.01% human serum albumin in RSM solution to 1/10 for dose level 1 and by 1/3 for dose level 2.

### Intratumoral injection of CalTUMP

Five to seven days after the end of PBT, a 1/10 dilution of CalTUMP was injected intradermally to test it for hypersensitivity and safety. Confirming that the skin induration was less than 2 cm after 48 hours, CalTUMP was injected directly into the tumor site under local anesthesia, guided by ultrasound imaging. Patients were injected a total of 3 times at 7-day intervals. As indicated in Table 1, each patient received 3 treatments with one of the 3 dose levels (1/10, 1/3 or 1/1) of CalTUMP. Each dose level was tested in 3 subjects each.

### Evaluation

The primary endpoint of this trial was to determine the safety of this combined treatment. Clinical symptoms and laboratory data were evaluated in accordance with the Common Terminology Criteria for Adverse Events (CTCAE v3.0) [29]. Blood and urine samples were collected before and after PBT, after the 3rd CalTUMP injection, 2 weeks later, and then every 2 months thereafter. Complete blood counts, serum levels of c-reactive protein (CRP), and urinalyses were followed. Tumor markers including lens culinaris agglutinin-reactive fraction of alpha-fetoprotein (AFP-L3 fraction), protein induced by vitamin K absence or antagonist (PIVKA)-II, carcinoembryonic antigen (CEA), pancreatic cancer associated antigen (DUPAN-2), carbohydrate antigen (CA) 19–9 were monitored, and ultrasounds were taken at these sampling points. Computed tomography (CT) of the lung and abdomen was conducted to exclude possible embolic or hemorrhagic complications on the day following the third CalTUMP injection. Tumor size was evaluated in accordance with the Response Evaluation Criteria in Solid Tumors (RECIST) guidelines [31].

Secondary endpoints were as follows: 1) Time to disease progression, 2) Cause specific survival, 3) Overall survival, 4) Quality of life as measured by Karnofsky Performance Scale (KPS) [32].

### Ethics

This clinical trial was approved by the Committee for Medical Ethics at the University Hospital of Tsukuba. Prior to initiating any procedure related to the protocol, written informed consent was obtained from every patient, and patient wishes and consent were respected in all instances. In addition, the Critical Path Research and Education Integrated Leading Center, University Hospital of Tsukuba accepted the monitoring of protocol compliance and patients’ status independently. We registered this study in the University Hospital Medical Information Network Clinical Trials Registry (UMIN-CTR) Japan (identification # 000002863, Tokyo).

## Results

**Figure 2:**
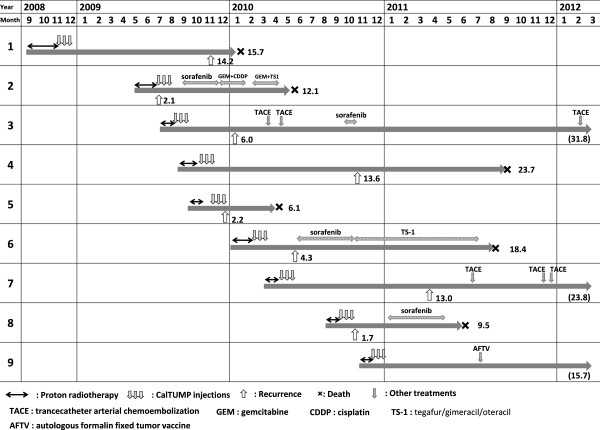
**Clinical courses of 9 patients.** Clinical courses of 9 cases are illustrated. Upward-arrows indicate recurrence or metastasis, and numbers indicate duration (months) after the initiation of PBT. Numbers at the right end of bars indicate follow-up duration in months by the end of March 2012.

Between September 2009 and December 2010, 9 patients (8 men and 1 woman) who met the inclusion criteria were enrolled in this clinical study as shown in Table 1. The entire clinical courses of these 9 patients are summarized in Figure 2. At the time of this analysis, 6 patients had died of HCC recurrence (#1,2,4,5,6,8), 2 patients were alive, but had recurrent tumors (#3, 7) and 1 patient was alive with no evidence of recurrence (#9).

### Safety

Every patient tolerated the treatment well and was able to complete the entire protocol with the exception of one patient (#5) in whom PBT was suspended at 52.8 GyE/16 fractions due to an increase in ascites. However, intratumoral injections of CalTUMP were performed as planned. In case #6, 15 GyE/5 fractions were boosted to the tumor at S5 to compensate for the 20% dose inadequacy in the initial treatment plan (Table 1). Acute toxicity related to PBT was observed in all patients: radiation dermatitis with G2, slight increases of SGOT and SGPT in one patient (#3), a temporary increase of bilirubin in one (#5), and a temporary increase of BUN and creatinine in one (#6). All laboratory data subsequently normalized without any clinical manifestations. As for the toxicity associated with intratumoral injections of CalTUMP, 7 patients (# 1, 2, 3, 4, 6, 7, 9) showed some low-grade fever between 37.1 and 38.4 as shown in Table 2. Laboratory data after injections showed a slight increase of CRP in 3 cases (#1,2,7), a decrease of PT in one (#5) and increases of SAST, SALT, γ-GTP, ALP and CRP in one (#9). Laboratory changes were temporary and required no medical intervention. No hemorrhagic or infectious complication related to treatment was noted. In addition, CT scans taken on the day after the final intratumoral injection, demonstrated no evidence of lung or abdominal complications. Although we escalated dose of CalTUMP from 1/10 to 1/1, there was no significant increase of adverse effects associated with this dose escalation.

**Table 2:** Acute toxicity and cause of death

**No**	**Age/gender**	**PBT dose/frac**	**Skin**	**Labo data-1**	**CalTUMP dose**	**Vital sign changes**	**Labo data-2**	**Cause of death**
1	73/M	74 GyE/37f	G2	n.p.	1/10	I. None	Slight increase of CRP	HF due to HCC progression
						II. 37.0-37.7 in 1–2 days		
						III. 37.1-37.5 in 1–2 days		
2	55/M	74 GyE/37 f	G2	n.p.	1/10	I. 37.4 in 1 day	Slight increase of CRP	HF due to HCC progression
						II. None		
						III. None		
3	52/M	76.2 GyE/22 f	G2	Slight increase of SGOT, SGPT	1/10	I. 37.1 in 1 day	n.p.	n.p.
						II. None		
						III. None		
4	63/M	76.2 GyE/22 f	G2	n.p.	1/3	I. None	n.p.	HF due to HCC progression
						II. None		
						III. 37.4-37.7 in 1–2 days		
5	65/M	52.8 GyE/16 f	G2	Increase of Bil	1/3	I. None	Decrease of PT	HF due to HCC progression
						II. None		
						III. None		
6	65/M	87.6 GyE/27 f	G2	Increase of BUN, creat	1/3	I. None	n.p.	HF due to HCC progression
						II. None		
						III. 37.4 in 1 day		
7	59/M	76.2 GyE/22 f	G2	n.p.	1/1	I. 37.4 - 38.0 in 1 day	Slight increase of CRP	n.p.
						II. 37.5 - 38.4 in 1 day		
						III. 37.7 in 1 day		
8	72/M	76.2 GyE/22 f	G2	n.p.	1/1	I. None	n.p.	HF due to HCC progression
						II. None		
						III. None		
9	71/F	76.2 GyE/22 f	G2	n.p.	1/1	I. 37.0 in 1 day	Increase of SGOT, SGPT, γ-GTP, ALP, CRP	n.p.
						II. 37.1 in 1 day		
						III. 37.8 on the same day		

(PBT: Proton beam radiotherapy, Dose/frac: Total dose/fraction number, Labo data-1: Laboratory data changes associated with PBT, Labo data-2: Laboratory data changes associated with CalTUMP injection, HF: Hepatic failure).

Acute toxicities associated with PBT and CalTUMP injections were listed. Cause of death in 6 patients who died during the follow-up periods were listed.

The direct cause of death in 6 patients was hepatic failure due to HCC progression. The three cases (#2,5,8) who died within 12 months of the final injection were critically reviewed by two independent safety and efficacy committee members. Both concluded that the deaths in these cases were due to HCC progression, and the causality of the treatment was deemed minimal or unrelated.

These results suggest that the toxicity associated with PBT was of the same level as previously reported [33]. In addition, the adverse effects of intratumoral injections of CalTUMP were shown to be within tolerable limits. However, maximum tolerant dose of CalTUMP was not determined as there was no significant correlation between occurrence of toxicity events and dose of CalTUMP.

### Efficacy

Recurrent disease was seen in one patient (#2) immediately after PBT and two patients (#5, #8) immediately after the third injection of CalTUMP. These three had significantly shorter survival times as compared to 6 other cases including 4 cases (#1,4,7,9) who were recurrence free for more than one year from the initiation of PBT (Figure 2). As there was no constraint on selection of treatment after recurrence, patients #3, 7 underwent multiple sessions of transarterial chemoembolization, patients #2, 6, 8 received chemotherapy consisting of either cisplatin, gemcitabine or tegafur/gimeracil/oteracil (TS-1), and patients #3, 8 were treated with sorafenib. Case #9 received AFTV [28] as per her wishes 7 months after the 3rd injection of CalTUMP without noticing recurrence, and has thus far shown no evidence of progression, and is currently stable.

## Discussion

In the present phase-I clinical trial, we could demonstrate that the direct intratumoral injection of the newly developed immunoadjuvant, CalTUMP, for inducing *in situ* tumor vaccination after PBT was both feasible and safe in patients with HCC. We are now planning a phase II study to extend these observations and further evaluate the efficacy of this approach.

Several strategies have been tried to prevent the recurrence of HCC. Among them, retinoic acid [34] and interferon-alpha [35] have shown efficacy in preventing HCC recurrence after surgical resection. We have previously reported in a randomized clinical trial that active immunotherapy using AFTV successfully prolonged both overall and progression-free survivals after surgical resection of HCC [27]. AFTV was also effective in patients with glioblastoma multiforme (GBM) prolonging survival periods to 19.8 months or more [36,37]. Although the sample sizes in these reports were small, the results were favorable as compared to the median overall survival of 14.6 months achieved by the GBM standard therapy consisting of primary resection, radiation therapy plus temozolomide administration [38].

However, a major drawback of AFTV is that it can be applied only to the patients who have at least 1.5 gram of surgically resected autologous tumor tissues. In order to overcome this limitation, we have developed an *in situ* vaccination in a mouse model, directly injecting a microparticulated cytokine immunoadjuvant into microwave-denatured established tumors [39]. The injected adjuvant not only suppressed local recurrence at the primary tumor site, but also tumor formation at a differently challenged site. Theoretically, it may be possible to induce a systemic immune response called abscopal effect when the AFTV immunoadjuvant is applied to the *in vivo* denatured tumor site, which can be referred to as *in situ* vaccination.

It has been reported that ionizing radiation up-regulated immunological cell surface molecules such as ICAM-1, CEA, and mucin-1 on human cancer cells in vitro [40,41]. In addition, we have previously reported that x-ray irradiation enhanced immunogenicity of tumor cells by up-regulation of molecules such as Fas and MHC-I in human brain tumor cells in vitro [42]. Furthermore, Apetoh et al. reported that radiation-induced cell death released high mobility group protein B1 that binds heat shock proteins to toll-like receptor (TLR)-4 on antigen-presenting dendritic cells [43]. These reports suggest that a combination of confocal radiotherapy and systemic immunotherapy is a promising way to induce synergistic effects on solid neoplasms. In particular, we have demonstrated that PBT is effective in the local control of HCC due to the excellent dose conformity to the target while preserving surrounding normal tissue [10,12-17]. These characteristics of PBT may suggest that PBT also has an advantage for preserving potential immune reaction in the local tumor environment.

Based on these, a strategy of *in situ* vaccination combining cytotoxic therapy and immunoactivation has been explored. Brody et al. reported that focal low dose radiotherapy to one of the tumor sites and injection of a TLR-9 agonist at the same site induced systemic tumor specific immune response and demonstrated complete or partial response in 4 of 15 patients with relapsed B-cell lymphoma [44]. Although this favorable result may be due to the nature of the high response rate of B-cell lymphoma to passive or active immunotherapy, they could demonstrate acceptable feasibility and efficacy of *in situ* vaccination in patients with relapsed B-cell lymphoma burden. In our study, when the composition of immunoadjuvant used for AFTV is delivered directly into the tumor tissue and bound to denaturing (apoptotic/necrotic) tumor cells after PBT, the immunoadjuvant-coated tumor cell fragments are expected to become an *in situ* tumor vaccine. Similar effects may possibly be expected for patients with HCC treated by local ablation, as necrotic or apoptotic tumor tissue left in situ are available as tumor antigens. However, this treatment is not suitable for patients undergoing surgery because the tumor tissue is removed.

Hydroxyapatite (HA; Ca_10_(PO_4_)_6_(OH)_2_) was used as a sparingly soluble carrier of BCG extract to maintain bioactivity at the injected site in this study. HA is a highly biocompatible material widely used as bone substitute since its chemical composition and crystal structure closely resemble bone mineral. Thus, it has been substantiated through clinical use as an osteoconductive bone substitute for over three decades [45]. Furthermore, a clinical pilot study was conducted using heated HA particles which had adsorbed a patient’s self-tumor antigens [46]. It is known that HA precipitated in an aqueous solution shows low crystallinity, large surface areas and high adsorption nature, while HA heated at high temperatures has high crystallinity. Calcium phosphate adjuvant precipitated in a solution with a Ca/P molar ratio of 1.0 has also been used in humans as an immunoadjuvant for many years [47]. And now, commercially available calcium phosphate adjuvant is HA with a Ca/P molar ratio between 1.67 and 1.33 [48]. It has been demonstrated that soluble bioactive molecules coprecipitated with HA in a supersaturated calcium phosphate solution is released from HA in a sustained manner and retain bioactivity [49,50].

Although we assumed that the percutaneous intratumoral injection of CalTUMP might be associated with some risks of adverse events such as hemorrhage, infection or needle track tumor seeding, we fortunately did not observe any of these serious complications either in examinations or laboratory data, indicating that this procedure is both feasible and safe. The incidence of needle tract tumor seeding associated with HCC biopsy has been reported to be 3.4 to 5.1% [51,52]. However, the incidence of seeding with our procedure would probably be much lower, as no aspiration for tumor sampling was performed, and centesis was done after the tumor was inactivated by high proton beam radiotherapy. Also, the safety of CalTUMP was tested by injecting three different doses (1/10, 1/3, 1/1). Although it was shown that all 3 doses were tolerable, the occurrence of adverse effects was not in a clear dose dependent manner. This might indicate that we could not disclose the maximum tolerant dose of CalTUMP in this study, and that adverse effects of the immunoadjuvant may not be evaluable by a simple dose escalation method. Further analysis is required.

**Figure 3:**
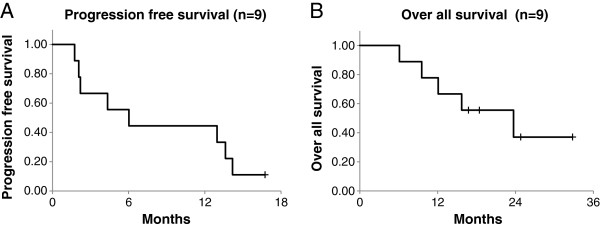
**Progression free (A) and overall (B) survival curves of the patients who underwent the protocol.** Survival curves were drawn by the Kaplan-Meier method.

With regard to the efficacy, we could not observe dramatic improvements in the outcome in some of our patients. It should be noted, however, that all patients in this trial had highly advanced diseases with histories of either multiple tumors or repeated recurrences. In 3 of the 9 patients, even extra-field liver or lymph node metastases were found on imaging examinations immediately after PBT or CalTUMP injections, strongly suggesting that minute recurrences had already been existed before CalTUMP injection was started. The clinical recurrence of HCC is in general composed of *de novo* carcinogenesis (true recurrence) and intra-hepatic metastasis. Therefore, in our next phase II trial aimed at examining the efficacy of this approach, we should use a better inclusion criteria which would allow us to select patients more suitable for examining the prevention effects of CalTUMP on the *de novo* carcinogenesis, such as those with the first and solitary recurrence of HCC, and to exclude patients likely to have already multiple metastatic tumors as some of the patients in this trial. Another possibility for the lack of dramatic improvement in the outcome is the lack of adequate immune response for those advanced stage patients, based on peripheral blood samples and vital signs, suggesting that the CalTUMP dose may have been insufficient. However, the doses of CalTUMP used in this trial did induce non-specific inflammatory responses such as transient fever or increased levels of c-reactive protein (CRP) in 7 of the patients (#1, 2, 3, 4, 6, 7, 9), and the lack of these responses in the remaining 2 (#5,8) was associated with a significantly poorer prognosis (Table 2, Figure 3). Actually, we also tried to evaluate the efficacy of this therapy by comparing overall and progression-free survival periods in a case-controlled analysis. Ten patients in the control group for the comparative analysis were in the same stage and condition as those receiving the combined therapy. Although the small sample sizes prevented us from drawing any solid conclusions, this comparative analysis did reveal a tendency for prolonged progression-free survival in patients receiving CalTUMP despite their median KPS being significantly lower than that of the control group. In this study, we simply examined the survival rate and time to progression from the last PBT followed by CalTUMP injection, regardless of the previous treatments performed before the last PBT. The genuine efficacy of this in situ vaccine for the reduction of HCC recurrence should be examined in the future by a randomized controlled trial with improved patient selection criteria, where the effects of all potential confounding factors, including previous treatments, can be eliminated through randomization.

In conclusion, direct intratumoral injection of CalTUMP after PBT in patients with HCC was found to be both feasible and safe. This approach is unique that it uses *in situ* inactivated tumor tissue and a newly-developed immunoadjuvant to induce *in situ* vaccination. Establishing the safety and efficacy of this therapeutic strategy may help prevent HCC recurrence and also prove effectiveness against other solid cancers.

## Competing interests

The authors declare that they have no competing interests.

## Authors’ contributions

KT, TO, AT designed and obtained funding for the study. YS, AI, TO made the immune adjuvant. TO, TH AK, HHN MM, TS, HS performed PBT. MA, TO, KF, TH, MA, KI acquired clinical data and performed analysis. MA, IH, AT, TO, KT made the interpretation of data. MA, JZ, GA, AI, TO, KT involved in drafting paper, tables and figures. All authors read and approved the final manuscript.
